# Assessment of the Influence of Crystalline Form on Cyto-Genotoxic and Inflammatory Effects Induced by TiO_2_ Nanoparticles on Human Bronchial and Alveolar Cells

**DOI:** 10.3390/nano11010253

**Published:** 2021-01-19

**Authors:** Anna Maria Fresegna, Cinzia Lucia Ursini, Aureliano Ciervo, Raffaele Maiello, Stefano Casciardi, Sergio Iavicoli, Delia Cavallo

**Affiliations:** Department of Occupational and Environmental Medicine, Epidemiology and Hygiene, Italian Workers’ Compensation Authority—INAIL, 00078 Monte Porzio Catone, Rome, Italy; a.fresegna@inail.it (A.M.F.); c.ursini@inail.it (C.L.U.); au.ciervo@inail.it (A.C.); r.maiello@inail.it (R.M.); s.casciardi@inail.it (S.C.); s.iavicoli@inail.it (S.I.)

**Keywords:** TiO_2_NP crystalline form, genotoxicity, cytotoxicity, inflammation, human lung cells

## Abstract

Titanium dioxide nanoparticles (TiO_2_NPs) are increasingly used in consumer products, industrial and medical applications, raising concerns on their potential toxicity. The available in vitro and in vivo studies on these NPs show controversial results. Crystalline structure is the physicochemical characteristic that seems to influence mainly TiO_2_NPs toxicity, so its effect needs to be further studied. We aimed to study whether and how crystalline form influences potential cyto-genotoxic and inflammatory effects induced by two commercial TiO_2_NPs (TiO_2_-A, mainly anatase; TiO_2_-B, mainly rutile) in human alveolar A549 and bronchial BEAS-2B cells exposed to 1–40 µg/mL. Cell viability (WST-1), membrane damage (LDH release), IL-6, IL-8 and TNF-α release (ELISA) and direct/oxidative DNA damage (fpg-comet assay) were evaluated. Physicochemical characterization included analysis of crystalline form (TEM and XRD), specific surface area (BET), agglomeration (DLS) and Z-potential (ELS). Our results show that TiO_2_-A NPs induce in BEAS-2B cytotoxicity and a slight inflammation and in A549 slight oxidative effects, whereas TiO_2_-B NPs induce genotoxic/oxidative effects in both cell lines, revealing different toxicity mechanisms for the two tested NPs. In conclusion, our study confirms the influence of crystalline form on cellular response, also demonstrating the suitability of our in vitro model to screen early TiO_2_NPs effects.

## 1. Introduction

Nano-Titanium dioxide (nano-TiO_2_) has become the most highly used nanoparticle (NP) worldwide [1], occurring in daily products such as paints, plastics, papers, inks, foods, pharmaceuticals, cosmetics [2] and many personal care products [3]. Thus, due to their widespread use and the consequent unintentional exposure, the concern about the potential toxicity of TiO_2_ nanoparticles (TiO_2_NPs) is raising. Exposure to TiO_2_ nanoparticles can occur during manufacturing, handling and use [4,5] involving consumers, workers, researchers of Laboratories involved in the development and production of nanomaterials (NMs) [6].

Titanium dioxide is the naturally occurring oxide of titanium and has different crystalline structures: rutile, the most common natural and stable form [1,7], anatase, the most commercially used type [4], brookite, the rarest form [7]; there is also a fourth amorphous form named “titanium dioxide (B)” [8]. In its nano-form, TiO_2_ belongs to the category of metallic NPs [9]. The International Agency for Research on Cancer (IARC) has reclassified TiO_2_ as a Group 2B carcinogen (possibly carcinogenic to humans) on the basis of inadequate evidence in humans and sufficient evidence in experimental animals that fine TiO_2_ particles cause cancer [8].

The respiratory tract is the main exposure target [10,11]: NPs can be inhaled, and thus enter the circulatory system resulting in systemic distribution, inflammation, and cardiovascular and neurological effects [12], as well as bioaccumulation in tissues and organs [13] and finally cancers [14].

To date, the available studies on the toxicity of TiO_2_NPs show controversial results [15], especially those about genotoxicity [16]: this contradiction maybe due to the different particle used, to cell culture media and cell systems, as well as to the testing methods [17].

TiO_2_NPs enter the cell via endocytosis [18] and induce oxidative stress causing an increase of the intracellular amount of reactive oxygen species (ROS) and depleting antioxidant defense, thus resulting, among other things, in local inflammation, mitochondria damage, cell autophagy, apoptosis, or necrosis [19]. In addition, oxidative stress can lead to indirect genotoxic effects [17], even if direct interaction with the DNA cannot be excluded [20], and to unregulated cell signaling, cytotoxicity, protein and lipid alterations, and epigenetic effects [21].

NM physicochemical characteristics including specific surface area, size, shape, crystal phase, chemical composition, charge or other surface characteristics could influence the pulmonary toxicity and inflammatory response [22]. The smaller size of NMs, associated to increased specific surface area can cause increased toxicity and translocation, greater lung retention, and slower pulmonary clearance [22].

It is well known that NPs exhibit different properties compared to bulk materials: given their high surface area to volume ratio, they are highly reactive [23], because the larger surface area provides a larger interface for interaction with biological fluids and tissues [22]. Existing studies suggest that TiO_2_NPs are more toxic than bulk TiO_2_ [19].

Moreover, crystalline structure is the physicochemical characteristic that seems to influence mainly TiO_2_NPs toxicity [22]. Several in vitro studies report that anatase titania induced oxidative stress, apoptosis, and mitochondrial impairment in human glial and lung cells and cytotoxicity in Chinese hamster fibroblasts and murine osteocells, whereas rutile titania exerted cytotoxic effects in human amnion and skin, in Chinese hamster fibroblasts but not in human intestinal cells, in nasal mucosa cells and in lymphocytes [24].

Despite the importance of the crystalline form, few studies, and discordant, have attempted to investigate its possible toxic effects [22]. Recently Danielsen et al. [22] have studied the pulmonary toxicity of four different anatase TiO_2_NPs in mice exposed by intratracheal instillation and compared it to that of rutile TiO_2_NPs from their previous study. They have found that rutile form was more inflammogenic in terms of increase of neutrophils than anatase, when normalized to total deposited surface area, so they have concluded that specific surface area, crystal phase, and shape of nano-TiO_2_ are important predictors for their observed pulmonary effects [22]. Vandebriel et al. [25] evaluated the effects of fourteen different TiO_2_NPs on dendritic cells (DC), part of the lung immune system, in mice exposed by intranasal treatment. They found that anatase nano-TiO_2_ induced a more strong DC maturation than rutile and showed a stronger adjuvant activity in an in vivo allergy model, so they suggested that, from the perspective of “safe by design”, rutile may be preferred over anatase TiO_2_NPs, especially when inhalation exposure can be expected during manufacturing or use of the product [25]. In a study performed by Yu et al. [26] a mouse macrophage line was exposed to anatase and rutile TiO_2_NPs in order to explore their toxicity. They found that the anatase NPs have a high affinity to proteins and impair mitochondrial function; in contrast, the rutile NPs have high affinity to phospholipids and target the membranes, causing a ROS-independent necrotic cell death; all thus implying that rutile TiO_2_NPs exert a slight higher toxicity than anatase [26]. Ghosh et al. [27] found that, in a human bronchial epithelial cell line exposed to different crystal phases of nano-TiO_2_, anatase form was more cytotoxic compared to the rutile and anatase-rutile mixture. Genotoxicity measured by comet assay also revealed higher toxicity of the anatase form, whereas the micronucleus assay did not reveal any significant difference between the crystal phases. Finally, all the crystal phases caused significant alteration in DNA methylation levels after 24 h exposure so, taking into account that global DNA hypomethylation has been associated with several diseases and cancer, the authors suggested that epigenetic endpoints should be considered in the safety assessment of TiO_2_NPs in various consumer products [27]. Uboldi et al. [24] in a study on mouse fibroblasts exposed to pure anatase and rutile TiO_2_NPs, evidenced only for rutile TiO_2_NPs a cytotoxic effect in terms of dose-dependent reduction of the clonogenic activity. Similarly, rutile nano-TiO_2_ appeared to be slightly clonogenic and genotoxic, whereas anatase TiO_2_NPs did not induce any significant neoplastic or genotoxic effect. Additionally, as indicated by the quantification of the in vitro uptake of TiO_2_NPs, they showed that the internalization was independent of the crystalline form but size dependent, as nano-TiO_2_ were taken up more than their respective bulk materials [24].

In the present work we aimed to study whether and how crystalline form influences potential cyto-genotoxic and inflammatory effects induced by two commercial TiO_2_NPs (A, 79% anatase; B, 81% rutile) in human lung cells. In particular, here we report the results obtained testing the rutile nano-form, TiO_2_-B, and compare them with those observed on the anatase crystalline form, TiO_2_-A, previously published [10]. To do this, we used the same well-characterized experimental approach (methods and cellular in vitro model) we developed to study the potential toxicity of other nanomaterials such as pristine and functionalized carbon nanotubes [28,29], and cobalt oxide NPs [30], in addition to the mentioned anatase titanium dioxide NPs [10]. The cellular model involves the use of human alveolar and bronchial epithelial cells (A549 and BEAS-2B) that represent the main lung cells and the most used cell types in inhalation toxicity studies [28].

## 2. Materials and Methods

### 2.1. Nanoparticles

Two commercially available titanium (IV) oxide nano-powders, product number 677,469 (TiO_2_-A) and product number 634,662 (TiO_2_-B), were purchased from Sigma-Aldrich (St. Louis, MO, USA). As specified by the supplier, the purity of TiO_2_-A was up to 97% and 1% Mn was present in the sample as dopant; the purity of TiO_2_-B was 99.5% with trace metals basis.

### 2.2. Nanoparticles Characterization

For characterization, we prepared a stock solution (2 mg/mL) of TiO_2_NPs in ultrapure sterile water, then we vortexed it for 1 min and sonicated for 5 min to disperse NPs.

NPs diameter and shape, and agglomerate sizes in water and cell culture media (RPMI-1640 and BEGM) were analyzed by an Energy Filtered Transmission Electron Microscopy (EFTEM) (FEI TECNAI 12G2 Twin; Termo Fisher Scientific-FEI Company, Hillsboro, OR, USA), at an accelerating voltage of 120 kV, equipped with an electron energy filter (Gatan Image Filter, BioFilter model; Gatan Inc., Pleasanton, CA, USA) and a Peltier cooled charge-coupled device based slow scan camera (Gatan multiscan camera, model 794IF; Gatan Inc., Pleasanton, CA, USA). We acquired conventional and High Resolution TEM (HRTEM) micrographs and performed the elemental and structural analysis by Electron Energy Loss Spectroscopy (EELS) and Nanobeam Electron Diffraction (NED). HRTEM, EELS and NED showed the crystalline nature of nanoparticles. The image analysis was performed by Digital Micrograph (Gatan Inc., Pleasanton, CA, USA) and ImageJ (National Institutes of Health, Bethesda, MD, USA).

NPs hydrodynamic diameter and zeta potential were analyzed by Dynamic Light Scattering (DLS) and Electrophoretic Light Scattering (ELS) (Zetasizer nano ZS, Malvern, UK). Suspension stability was evaluated measuring the hydrodynamic diameter of the TiO_2_NPs in each culture medium at the beginning of exposure (t0) and after 24 h (t24).

The Specific Surface Area (SSA) of samples was obtained by N_2_ adsorption at 77 K using the Brunauer–Emmett–Teller (BET) method and a Quantachrome Nova 2200 Surface Area Analyzer (Quantachrome Instruments, Boynton Beach, FL, USA).

The crystalline phase was analyzed by X-ray diffraction (XRD), using a simultaneous 120° angular dispersion X-ray diffractometer (Italstructures, Novara, Italy; curved PSD detector from INEL, Artenay, France), equipped with a Fe Kα1 source. The phases amount and the grain size dimensions were evaluated by Rietveld refinement [31,32] using a MAUD-Material Analysis Using Diffraction software (Università di Trento, Trento, Italy).

### 2.3. Cell Culture

The human lung epithelial (A549) and bronchial epithelial (BEAS-2B) cell lines were obtained from American Type Culture Collection (ATCC) (Rockville, MD, USA). A549 cells were cultured in complete RPMI-1640 (EuroClone, Milan, Italy) supplemented with 10% heat-inactivated Fetal Bovine Serum (FBS; Sigma-Aldrich, St. Louis, MO, USA); BEAS-2B cells were cultured in BEGM (Cambrex Bio Science Walkersville Inc., East Rutherford, NJ, USA).

Cells were seeded into a 24 well culture plate (15.6 mm well diameter) at a density of 8 × 10^4^ cells per well and were grown for 24 h before exposure at 37 °C in 5% CO_2_ and humidified atmosphere. On the day of exposure, cell media were replaced with fresh media.

### 2.4. Exposure

At the time of exposure, the stock solutions in water (2 mg/mL) were vortexed 1 min and then sonicated 5 min (Branson 2510; Branson Ultrasonics Corporation, Danbury, CT, USA) to better disperse the NPs. Then, a working solution (1 mg/mL) in complete culture medium (RPMI-1640 or BEGM) was prepared and sonicated in two 5-min steps with a 30-s pause, before being quickly added to the wells to the final concentrations of 1, 5, 10, 20, and 40 μg/mL. During exposure, cells were maintained at 37 °C in 5% CO_2_ and humidified atmosphere. Three independent experiments were conducted in three separate times.

### 2.5. Cell Viability

The cell viability of cells exposed for 24 h to TiO_2_NPs was assessed by the colorimetric Premix WST-1 Cell Proliferation Assay System (Takara Bio Inc., Shiga, Japan). The WST-1 is assumed to be reliable to evaluate NM cytotoxicity since does not show interference with NPs including TiO_2_ as previously reported [33,34].

WST-1 is a water-soluble tetrazolium salt that is reduced by mitochondrial dehydrogenases in cells to a water-soluble formazan dye. The amount of the formazan dye is directly proportional to the number of living cells.

The assay was performed according to our previous study [10]. Briefly, after exposure, the media were removed and cells were rinsed twice with Phosphate Buffered Saline (PBS) to avoid any possible interference in light absorption due to TiO_2_NPs. Then, according to the manufacturer’s guidelines, fresh culture medium plus PreMix WST-1 (10:1 ratio) were added to each well and plates were incubated for 3 h at 37 °C in the dark. At the end of incubation, 200 μL of supernatant in duplicate were transferred in an optically clear 96-well flat bottom plate and absorbance [A] was measured at 450 nm using a spectrophotometric microplate reader (Wallac Victor 2; Perkin Elmer, Boston, MA, USA). Unexposed cells were used as negative control and cells exposed to 1% Triton X-100 (Sigma-Aldrich, St. Louis, MO, USA) were used as positive control. A blank well was set up with only fresh medium plus Premix WST-1 (10:1 ratio) and used as background. After subtracting background absorbance, the cell viability (% of control) was calculated as follows:

(1) $$\%\;\mathrm{viable}\;\mathrm{cells}=\frac{\left[A\right]\;\mathrm{sample}}{\left[A\right]\;\mathrm{negative}\;\mathrm{control}}\times 100.$$

### 2.6. Membrane Damage

To determine the membrane damage of cells exposed for 30 min, 2 h and 24 h to TiO_2_NPs, the lactate dehydrogenase (LDH) assay (Cytotoxicity Detection Kit; Roche Diagnostics, Mannheim, Germany) was used.

LDH assay is a colorimetric test performed to quantify cell membrane damage and is based on the reduction of tetrazolium salt to formazan by the LDH. The LDH is an enzyme present in all cells and it is rapidly released from the cytosol of damaged cells into the cell culture supernatant.

This assay was performed as previously [10], according to the manufacturer’s guidelines. Briefly, the reaction mixture plus the culture supernatant (1:1 ratio) were transferred in triplicate to the wells of an optically clear 96-well flat bottom plate and incubated for 30 min at T amb (15–25 °C), protecting the plate from light. Blank (only culture medium), negative controls (cells not exposed) and positive controls (cells exposed for the same exposure times to 1% Triton X-100) (Sigma-Aldrich, St. Louis, MO, USA) were arranged. At the end of incubation, absorbance was measured at 490 nm using a spectrophotometric microplate reader (iMark; Bio-Rad, Milan, Italy).

To take into account the possible interference in light absorption due to TiO_2_NPs, a parallel set of experiments was carried out without cells; then, the absorbance of each concentration of TiO_2_NPs (without cells) was subtracted from that of the corresponding sample (with cells).

Finally, after subtracting background absorbance, the percentage of cytotoxicity was calculated as follows:

(2) $$\%\;\mathrm{cytotoxicity}=\frac{\left(\mathrm{sample}-\mathrm{negative}\;\mathrm{control}\right)}{\left(\mathrm{positive}\;\mathrm{control}-\mathrm{negative}\;\mathrm{control}\right)}\times 100.$$

### 2.7. DNA Damage

To assess the genotoxic/oxidative DNA damage of cells exposed for 2 and 24 h to TiO_2_NPs we used the Fpg (Formamido-pyrimidine DNA glycosylase; Sigma-Aldrich, USA) modified comet assay which allows to evaluate direct and oxidative DNA damage simultaneously.

The Fpg is a DNA base excision repair enzyme that removes the oxidized guanines and some alkylated bases, generating strand breaks, and is used to detect the oxidative damage by comet assay [35].

The previously described protocol [36] was used. Briefly, after exposure, the cell suspensions from each experimental point are mixed with agarose and set on two Gelbond films (Sigma-Aldrich, USA) to evaluate direct and oxidative DNA damage at the same time. Unexposed cells are used as negative control and cells exposed for 30 min to 100 µM hydrogen peroxide (H_2_O_2_) are used as positive control. After lysis, one set of Gelbond films is incubated with Fpg enzyme to evaluate oxidative DNA damage and the other one only with buffer to detect direct DNA damage. This is followed by an electrophoresis in alkaline buffer performed in the dark. It is important to avoid any further DNA damage due to the possible photo-activation of TiO_2_NPs by laboratory light exposure as reported for P25 TiO_2_NPs by Petersen et al. (2014) who suggest to perform comet assay of TiO_2_ exposed cells in the dark to avoid potential artifacts [37].

After ethidium bromide staining, 100 randomly chosen comet images per slide are acquired by fluorescence microscopy (Axioplan 2 Imaging; Carl Zeiss, Göttingen, Germany) at 200× magnification and then analyzed by a specific image analysis software (Delta Sistemi, Rome, Italy).

DNA damage is calculated as the mean % tail DNA from 100 comets. For each experimental point, direct DNA damage is calculated, in comets after buffer incubation, as the ratio of % tail DNA of exposed cells to % tail DNA of negative control; oxidative DNA damage (Fpg-sensitive sites) is calculated by subtracting the % tail DNA with buffer incubation from the % tail DNA with enzyme incubation.

### 2.8. Detection of Cytokines

The release of interleukine 6 (IL-6), interleukine 8 (IL-8), and tumor necrosis factor-alpha (TNF-α) after 2 and 24 h exposure to TiO_2_NPs was quantified by Enzyme-Linked ImmunoSorbent Assay (ELISA) (eBioscience, Wien, Austria).

The cytokines IL-6, IL-8 and TNF-α represent important mediators of inflammation in humans; in particular, they have a pro-inflammatory effect, promoting inflammation process.

The manufacturer’s guidelines were followed. Briefly, after exposure, the cell supernatant was collected and stored at −20 °C until use. Samples, including negative controls (cells not exposed to TiO_2_NPs), appropriate cytokine standards, blank and control samples were tested in duplicate. The absorbance was measured at 450 nm and quantified with a microplate absorbance reader (iMark; Bio-Rad, Milan, Italy).

### 2.9. Statistical Analysis

Three independent experiments were conducted and data are expressed as mean ± standard deviation (SD). Statistical significance of the data was analyzed by Non-parametric Kruskal–Wallis test followed by post-hoc analysis via the Bonferroni correction or Dunnett’s T3 test. *p*-values ≤ 0.05 and ≤0.01 were considered significant.

## 3. Results

### 3.1. Nanoparticle Characterization

Table 1 shows the physicochemical characteristics of TiO_2_-A and TiO_2_-B and the testing methods used.

TEM and XRD analyses revealed that TiO_2_-A is mainly made up of anatase with a spherical shape, whereas TiO_2_-B is mainly in the rutile crystalline form with irregular-tetragonal shape. The tested NPs have similar SSA values but slightly different primary NP diameters, with TiO_2_-A diameter smaller than TiO_2_-B.

**Figure 1:**
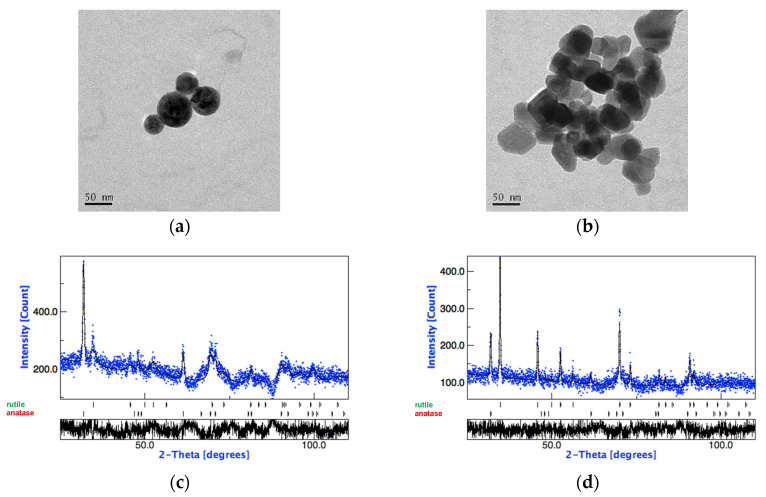
(**a**) TEM micrographs of TiO_2_-A and (**b**) TiO_2_-B NPs dispersed in water and deposited on a 300-mesh Cu grid coated with carbon film (bar 50 nm). (**c**) XRD pattern of TiO_2_-A: anatase: 79% and φ ≈ 45 nm, rutile: 21% and φ ≈ 6 nm; (**d**) XRD pattern of TiO_2_-B: anatase: 19% and φ ≈ 87 nm, rutile: 81% and φ ≈ 75 nm. Panel (**c**) related to TiO_2_-A has been previously published in [10] (Reproduced with permission from [10]. Copyright John Wiley & Sons, Ltd., 2014).

Figure 1 shows micrographs of TiO_2_-A and TiO_2_-B NPs obtained by TEM analysis and their XRD patterns.

High Resolution TEM (HRTEM), elemental and structural analysis by Electron Energy Loss Spectroscopy (EELS) and Nanobeam Electron Diffraction (NED) of TiO_2_-A were previously reported [10].

**Figure 2:**
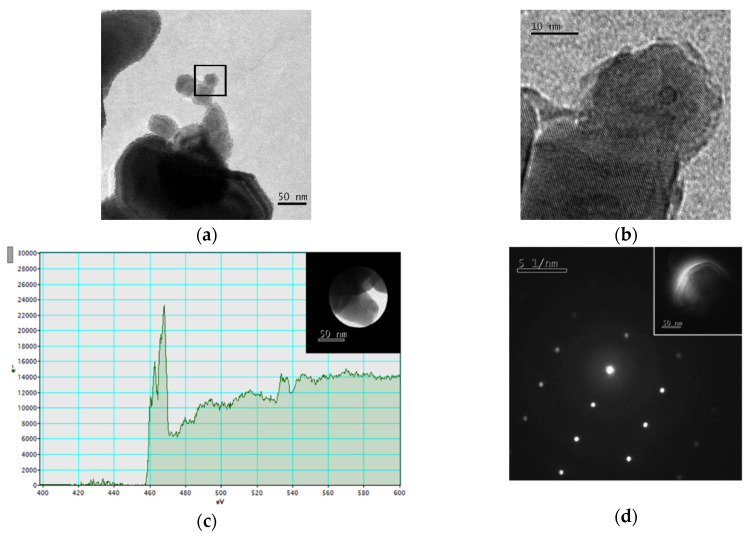
TEM TiO_2_-B NPs characterization. (**a**) TEM micrograph of TiO_2_-B NPs dispersed in water and deposited on a 300-mesh Cu grid coated with carbon film (bar 50 nm); (**b**) HRTEM image which reveals lattice planes of the particles inside the box marked in panel (**a**); (**c**) the EELS spectrum of NPs shown in the inset shows the peaks of the Ti-L_2,3_ thresholds (461 eV and 455 eV) and of the O-K threshold (532 eV); (**d**) Electron nanodiffraction pattern of the particle shown in the inset.

The crystalline nature of the TiO_2_-B NPs was confirmed by the following TEM techniques: HRTEM image (Figure 2a,b) revealed the lattice planes allowing the measurement of the plane distance (0.36 nm); elemental analysis of the TiO_2_-B NPs by EELS (Figure 2c) showed the splitting of the titanium L_2_ and L_3_ core loss peaks, not observable in amorphous Ti; NED revealed the very regular diffraction spot pattern typical of rutile crystalline form (Figure 2d).

In both water and the culture media the TiO_2_NPs dissolved forming agglomerate/aggregate with Zav diameters smaller in TiO_2_-A than in TiO_2_-B. The agglomerate/aggregate sizes are greater in BEGM medium than in RPMI and in culture media than in water for both TiO_2_. DLS analysis shows a Z potential slightly more negative in BEGM than in RPMI and a much more negative value in water than in culture media for both TiO_2_ (Table 1). The agglomerate/aggregate sizes of TiO_2_-B in water and BEGM decrease over time (t0–t24) due to the sedimentation of large agglomerates. The agglomerate/aggregate sizes of TiO_2_-A, showed in Table 1, remain almost similar over time (t0–t24), suggesting suspension stability and low sedimentation.

### 3.2. Cell Viability (24 h)

**Figure 3:**
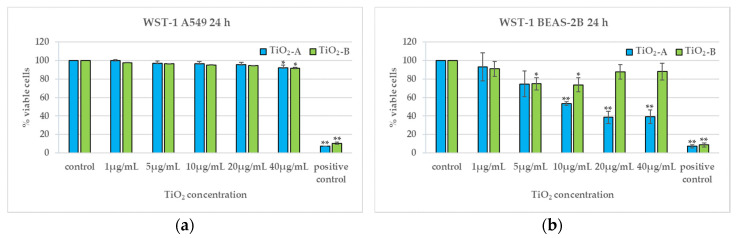
Viability percentage of A549 (**a**) and BEAS-2B (**b**) cells after 24 h exposure to TiO_2_NPs evaluated by the WST-1 assay. * *p* ≤ 0.05, ** *p* < 0.01. Data relative to TiO_2_-A are previously published in [10] (Reproduced with permission from [10]. Copyright John Wiley & Sons, Ltd., 2014).

Figure 3 shows the results of WST-1 assay and demonstrates that in A549 cells both TiO_2_NPs do not induce any cytotoxic effect, although there is a very slight viability reduction at the highest concentration (Figure 3a). In BEAS-2B cells, viability decreases differently for the two titania: TiO_2_-A NPs induce a dose-dependent viability reduction from 10 μg/mL; TiO_2_-B NPs induce a moderate viability reduction at 5 μg/mL and 10 μg/mL (Figure 3b).

### 3.3. Membrane Integrity (30 min, 2 h and 24 h)

**Figure 4:**
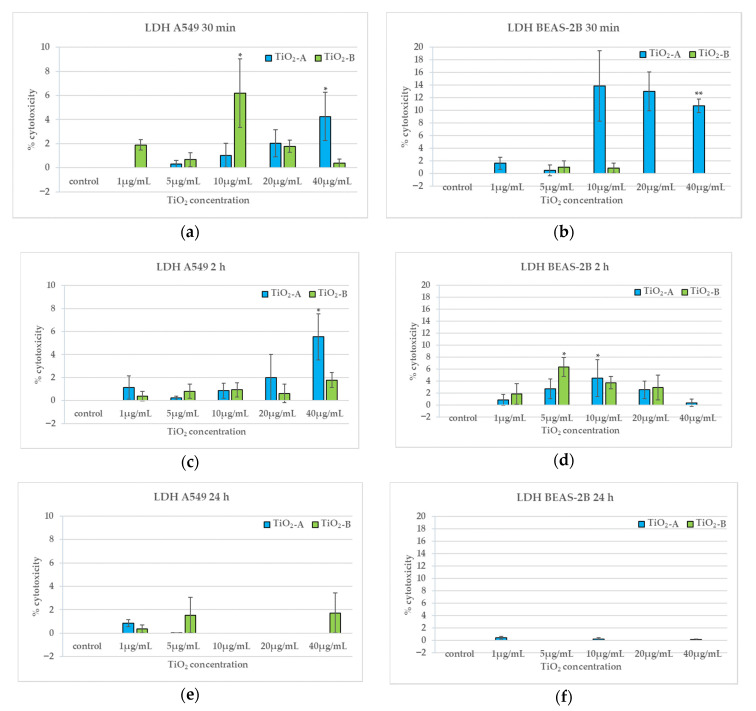
Lactate dehydrogenase (LDH) release expressed as a per cent of cytotoxicity in A549 and BEAS-2B cells after 30 min (**a**,**b**), 2 h (**c**,**d**) and 24 h (**e**,**f**) exposure to TiO_2_NPs. * *p* < 0.05, ** *p* < 0.01. Data relative to TiO_2_-A are previously published in [10] (Reproduced with permission from [10]. Copyright John Wiley & Sons, Ltd., 2014).

Figure 4 shows the results of LDH assay performed after 30 min, 2 h, and 24 h of exposure of A549 and BEAS-2B cells to the NPs. Both TiO_2_NPs induce a slight, early and transient increase of LDH release in both cell types. Furthermore, we found no effect on membrane integrity after 24 h exposure (Figure 4e,f).

In particular, TiO_2_-A NPs induce an increase in cytotoxicity at the highest concentration after 30 min and 2 h exposure in A549 (Figure 4a,c); in BEAS-2B TiO_2_-A induces a LDH release from 10 μg/mL, but statistically significant only at the highest concentration, after 30 min exposure and a peak at 10 μg/mL after 2 h exposure (Figure 4b,d). TiO_2_-B NPs causes a peak of LDH release after 30 min exposure at 10 μg/mL in A549 (Figure 4a) and after 2 h exposure at 5 μg/mL in BEAS-2B (Figure 4c).

### 3.4. DNA Damage (2 h and 24 h)

**Figure 5:**
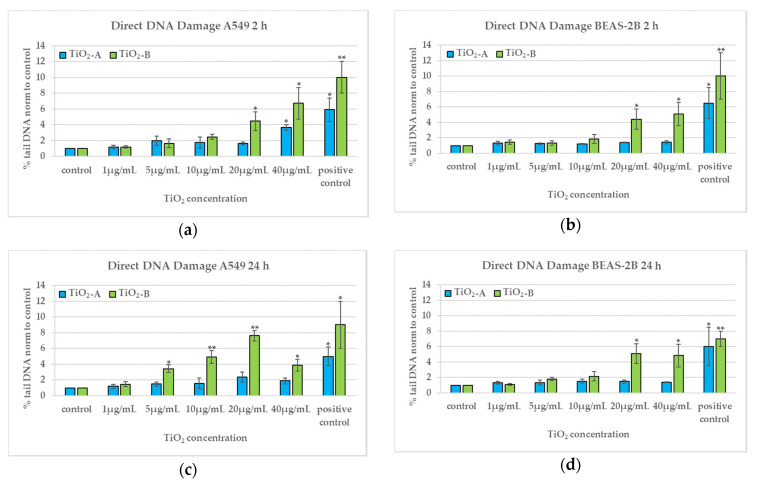

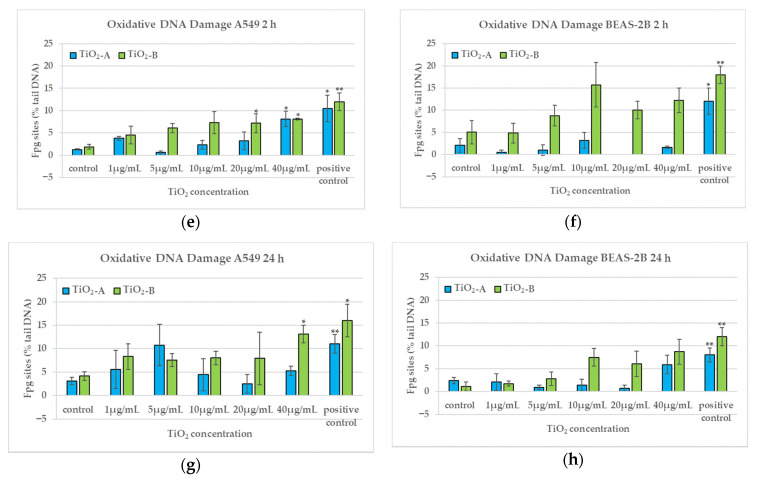
Direct and oxidative DNA damage in A549 and BEAS-2B cells after 2 h (**a**,**b**,**e**,**f**) and 24 h (**c**,**d**,**g**,**h**) exposure to TiO_2_NPs evaluated by the Fpg-comet test. * *p* < 0.05, ** *p* < 0.01. Data relative to TiO_2_-A are previously published in [10] (Reproduced with permission from [10]. Copyright John Wiley & Sons, Ltd., 2014).

TiO_2_-A NPs induce direct and oxidative DNA damage only after 2 h exposure at 40 µg/mL in A549 (Figure 5a,b,e,f); they induce neither direct nor oxidative DNA damage in both cell types after 24 h exposure (Figure 5c,d,g,h). On the other hand, TiO_2_-B NPs induce direct DNA damage after 2 h exposure at the highest concentrations in both cell types and an oxidative DNA damage at the highest concentrations statistically significant only in A549 (Figure 5a,b,e,f). After 24 h exposure, the direct DNA damage induced by TiO_2_-B is dose dependent from 5 µg/mL, with a peak at 20 µg/mL, in A549 cells and at the highest concentrations in BEAS-2B cells (Figure 5c,d). The oxidative DNA damage induced by TiO_2_-B after 24 h exposure is statistically significant only at the highest concentration in A549 cells (Figure 5g,h), whereas in BEAS-2B cells increased slightly starting from 10 µg/mL (Figure 5f).

### 3.5. Cytokine Release (2 h and 24 h)

**Figure 6:**
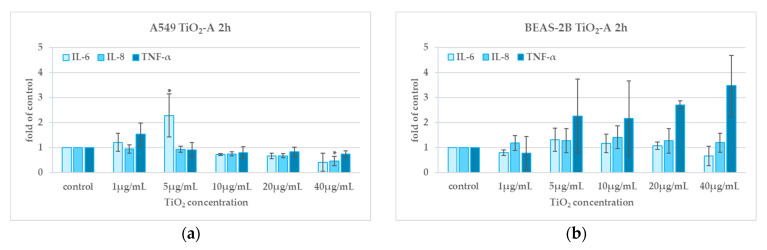

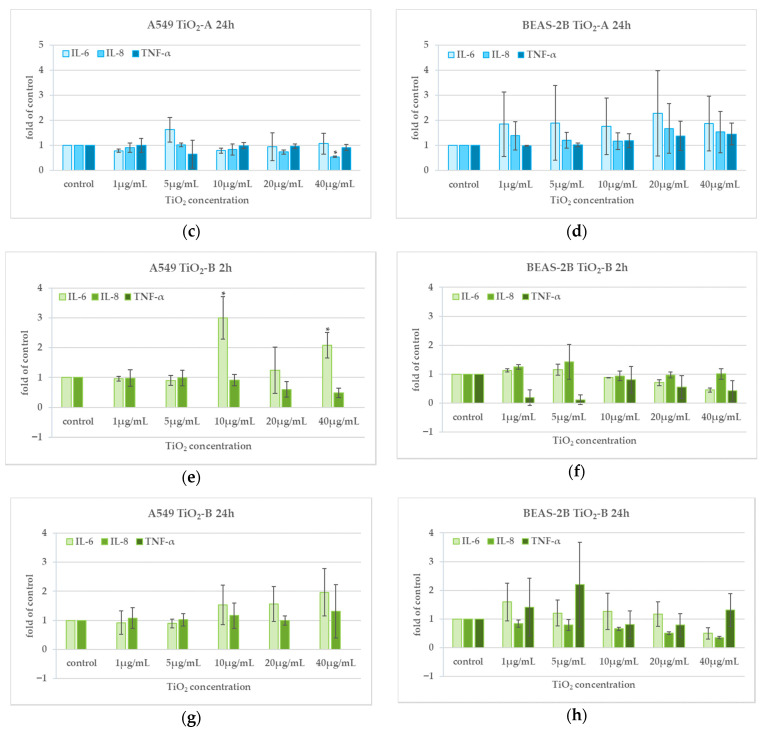
Cytokine release after 2 h (**a**,**b**,**e**,**f**) and 24 h (**c**,**d**,**g**,**h**) exposure to TiO_2_NPs in A549 and BEAS-2B cells. * *p* ≤ 0.05. Data relative to TiO_2_-A are previously published in [10] (Reproduced with permission from [10]. Copyright John Wiley & Sons, Ltd., 2014).

TiO_2_-A NPs induced in A549, a significant increase in the release of IL-6 at 5 µg/mL after 2 h exposure and a decrease in the release of IL-8 at the highest concentration after 2 h and 24 h exposure; we found no effect on the release of TNF-α after 2 h and 24 h exposure (Figure 6a,c). No statistically significant increase of cytokine release was detected in BEAS-2B (Figure 6b,d).

TiO_2_-B NPs induced an increase of IL-6 release in A549 cells after 2 h exposure at 10 and 40 µg/mL whereas no statistically significant increase of release of any of the three cytokines tested was found in BEAS-2B cells (Figure 6e–h).

## 4. Discussion

The toxicity of TiO_2_NPs has been evaluated in numerous in vitro and in vivo studies. Although anatase and rutile crystalline forms, which have different electrical and optical properties, are used in different applications, their potential ability to induce more or less toxicity still needs to be clarified. The final aim of this study was to understand if and how the crystalline form influences the potential toxicity of TiO_2_NPs on lung that represents one of the main target organs of occupational exposure to TiO_2_NPs. With this purpose, we used two types of commercial TiO_2_NPs that differed mainly for their crystalline form and we compared their potential ability to induce cytotoxic, genotoxic, and inflammatory effects on human alveolar and bronchial cells. TiO_2_-A exposure results, have already been published by our Laboratory [10], therefore in this work we compare the results of that study with those obtained testing rutile form TiO_2_-B.

The findings, summarised in Table 2, show the different toxic effects of the tested NPs, with high cytotoxicity, in terms of viability reduction, for TiO_2_-A in BEAS-2B cells, which responded to the injury of this kind of NPs with a dose-dependent trend, reaching about 40% of viable cells at the highest concentrations; differently, TiO_2_-B induced a lower decrease of cell viability in the same cell line.

This particular result confirms the higher susceptibility of bronchial BEAS-2B cells to cytotoxic effects and highlights the need to use more than one kind of cells when we have to screen the toxicity of new materials such as nanomaterials. TiO_2_-A induced also very early membrane damage in both cell lines, higher in BEAS-2B cells, which seems to correlate with the higher effects on cell viability. TiO_2_-B induced earlier membrane damage in A549 than in BEAS-2B cells whose membranes resulted damaged only after 2 h exposure. This different behaviour in membrane damage induction and consequently in cell viability reduction could be explained by the specific physicochemical characteristics (crystalline form, spherical shape and smaller NP diameter) that influence the lower tendency of TiO_2_-A compared to TiO_2_-B to agglomerate, with consequent smaller agglomerates that may cross directly and faster the membrane damaging it. Instead, larger TiO_2_-B agglomerates could enter the cell more slowly by an endocytosis mechanism.

The comparison between the two tested NPs on the induced genotoxic effects demonstrated that TiO_2_-A was able to induce early direct DNA damage only in A549 cells at the highest concentration, whereas TiO_2_-B induced DNA damage, after 2 and 24 h exposure, in both the tested cell lines. Differently, oxidative DNA damage was induced only in A549 cells after 2 h of exposure for TiO_2_-A and at both the times of exposure for TiO_2_-B. The lower genotoxicity of TiO_2_-A in respect to TiO_2_-B could be explained by the fact that uptaken agglomerates of TiO_2_ -A NPs, entered the cells through non-endocytic pathways, are too large to cross nucleus membrane and directly damage DNA. A small TiO_2_-A NPs fraction (the smallest agglomerates) could co-localize in the nuclei and other organelles, such as mitochondria, and induce genotoxic/oxidative effects. Therefore, only at the highest exposure concentration the TiO_2_-A NPs are in sufficient amount to induce early DNA damage in more susceptible A549 cells as previously hypothesized [10].

Both tested TiO_2_NPs induced an early and transient IL-6 cytokine release only in A549 cells that seems to correlate with the oxidative DNA damage found after 2 h exposure.

The analysis of agglomeration of the tested NPs performed by both TEM and DLS demonstrated very larger agglomerates of TiO_2_-B compared to those of TiO_2_-A in both culture media at the concentrations used to expose A549 and BEAS-2B cells. In addition, in BEGM medium used for BEAS-2B cultures, TiO_2_-B aggregates resulted larger than in the RPMI medium used for A549 cultures. These differences could explain the observed toxic effects. As reported by Veranth et al. [38], the tendency to agglomerate and sediment, also determined by the composition of specific culture medium and by the presence of serum proteins (as in complete RPMI), influences the biological effects, as NMs agglomerates sediment more easily and interact with the cells more rapidly and at higher effective concentrations than the non-agglomerated NMs [39,40]. It leads to greater toxic effects. The higher sedimentation of larger TiO_2_-B NP agglomerates could so explain the higher genotoxicity of rutile form in respect to anatase. The DNA damage induced by TiO_2_-B could be generated through an indirect mode of action probably through intra-cytoplasmatic oxidants induction linked to the higher photocatalytic properties of rutile TiO_2_NPs that make it able to generate ROS on their surface, as also reported by Fenoglio et al. [41]. The ability to induce genotoxic effects by ROS induction has been demonstrated for several kind of NPs [42]. However, other physicochemical properties such as size, mono/polydispersity or surface charge could play a synergistic role in the differences between the rutile and anatase nano-TiO_2_ toxicity mechanisms [24].

To date it is very difficult to find more than one study performed on the same NPs and an experimental protocol useful to make a comparison of the obtained results, therefore there is still a lack of information about the potential toxicity of NMs.

The comparison of the cytotoxicity of anatase and rutile form of TiO_2_NPs performed by Sayes et al. [43] on A549 cells, demonstrated a higher cytotoxicity for anatase compared to rutile and an increased release of IL-8 at concentrations higher than those used in our study. Higher toxicity of anatase compared to rutile in A549 cells was reported by De Matteis et al. [44], who also demonstrated the different degradation of the two crystalline forms in different conditions of pH and sunlight exposure. The Authors concluded that the higher toxicity of anatase is due to its major ionization.

Yu et al. [26] found a lower toxicity of anatase compared to rutile NPs in terms of viability reduction on macrophages. Uboldi et al. [24] demonstrated that rutile titania was slightly more toxic than anatase on Balb 3T3/mouse fibroblasts. Falck et al. [45] demonstrated that anatase reduces cell viability more than rutile on BEAS-2B cells.

The higher susceptibility of A549 cells to genotoxic effects induced by TiO_2_-B NPs, found in the present study and evident after 24 h exposure already at low concentrations, confirms what we found in our previous study for TiO_2_-A NPs [10] and in our other previous studies using the same experimental model [30,46]. The transformed A549 cells react to NM injury by direct and oxidative DNA damage because these cells are unaffected by cytotoxicity and more susceptible to genotoxic effects, probably because of lower capability to repair DNA damage by inactivation of both NER (Nucleotide Excision Repair) and BER (Base Excision Repair) [47].

Bronchial epithelial cells represent the first defence against airborne particulate matter and it may explain their stronger cytotoxic response in comparison with alveolar cells [48]. Normal BEAS-2B cells, respond to injury killing damaged cells (probably by apoptosis as reported by Park et al. 2008 [49] and Shi et al. 2010 [50]).

The different sensitivity of A549 and BEAS-2B cells suggests the need to use both cell types together with several and complementary toxicity end-points to evaluate the NM-induced biological effects.

Moreover, we highlight the need to evaluate the dispersion of tested NMs in the specific culture media. All together these factors contribute to induce the toxicity of NMs.

In conclusion, our findings confirm the influence of physicochemical characteristics and particularly of the crystalline form on cellular response to TiO_2_NPs, particularly regarding cytotoxic, genotoxic/oxidative effects and inflammation, with higher cytotoxicity for anatase exposure compared to rutile on bronchial cells and with rutile more genotoxic than anatase on both alveolar and bronchial cells. In addition, this study shows the suitability of our experimental system to screen toxic effects of nanomaterials with different properties.

## Figures and Tables

**Table 1:** Basic properties of the tested TiO_2_NPs.

**Primary Physicochemical Characteristics**	**TiO_2_-A** **Code 677469**	**TiO_2_-B** **Code 634662**	**Testing Method ^1^**
diameter (nm)	mean 43.8 ± 17.0 range 13.5–90.6	mean 76.6 ± 52.7 range 22.3–252.9	TEM
crystal structure	anatase (79%) rutile (21%)	rutile (81%) anatase (19%)	XRD
SSA (m^2^/g)	14.9	13.7	BET
shape	spherical	irregular–tetragonal	TEM
**Physicochemical characteristics of dispersed NPs in water (20 µg/mL)**	**TiO_2_-A** **Code 677469**	**TiO_2_-B** **Code 634662**	**Testing Method ^1^**
Z Potential (mV)	−32.2	−30.1	ELS
agglomerate/aggregate size (diam Zav nm)	139.8 (t0) 130.5 (t24)	366.0 (t0) 195.0 (t24)	DLS
**Physicochemical characteristics of dispersed NPs in RPMI with 10% FBS**	**TiO_2_-A** **Code 677469**	**TiO_2_-B** **Code 634662**	**Testing Method ^1^**
Z Potential (mV) (20 µg/mL)	−9.13	−10.3	ELS
agglomerate/aggregate size (diam Zav nm) (20 µg/mL)	196.5 (t0) 197.6 (t24)	368.0 (t0)	DLS
agglomerate/aggregate size (diam Zav nm) (100 µg/mL)	151.0 (t0)	447.0 (t0)	TEM
**Physicochemical characteristics of dispersed NPs in BEGM**	**TiO_2_-A** **Code 677469**	**TiO_2_-B** **Code 634662**	**Testing Method ^1^**
Z Potential (mV) (20 µg/mL)	−11.7	−11.0	ELS
agglomerate/aggregate size (diam Zav nm) (20 µg/mL)	220.2 (t0) 224.6 (t24)	428.0 (t0) 282.0 (t24)	DLS
agglomerate/aggregate size (diam Zav nm) (100 µg/mL)	186.0 (t0)	456.0 (t0)	TEM

^1^ Abbreviations: TEM, Transmission Electron Microscopy; XRD, X-Ray Diffraction; BET, Brunauer-Emmett-Teller; SSA, Specific Surface Area; DLS, Dynamic Light Scattering; ELS, Electrophoretic Light Scattering; diam Zav, diameter Z average.

**Table 2:** Cellular response induced by exposure to TiO_2_-A and TiO_2_-B.

Biological Endpoint	TiO_2_-A		TiO_2_-B	
	A549	BEAS-2B	A549	BEAS-2B
**Cell viability reduction**	+	+++	+	+
**Membrane damage**				
30 min	++	+	+	−
2 h	+	+	−	+
24 h	−	−	−	−
**Direct DNA damage**				
2 h	+	−	+++	+++
24 h	−	−	+++	+++
**Oxidative DNA damage**				
2 h	+	−	+++	−
24 h	−	−	+	−
**IL-6 release**				
2 h	+	−	+	−
24 h	−	−	−	−
**IL-8 release**				
2 h	+	−	−	−
24 h	+	−	−	−
**TNF-α release**				
2 h	−	−	−	−
24 h	−	−	−	−

+++, POSITIVE—Significant dose-dependent effect, with at least 2 statistically significant doses; ++, POSITIVE—Significant dose-dependent effect, with statistically significant highest dose; +, POSITIVE—No significant dose-dependent effect, with at least 1 statistically significant dose; −, NEGATIVE.

## Data Availability

The data presented in this study are available within this article. Further inquiries may be directed to the authors.

## References

[1] Zhang X., Li W., Yang Z. (2015). Toxicology of nanosized titanium dioxide: An update. Arch. Toxicol..

[2] Doak S.H., Dusinska M. (2017). NanoGenotoxicology: Present and the future. Mutagenesis.

[3] Weir A., Westerhoff P., Fabricius L., Hristovski K., von Goetz N. (2012). Titanium dioxide nanoparticles in food and personal care products. Environ. Sci. Technol..

[4] Bhattacharya K., Davoren M., Boertz J., Schins R.P., Hoffmann E., Dopp E. (2009). Titanium dioxide nanoparticles induce oxidative stress and DNA-adduct formation but not DNA-breakage in human lung cells. Part. Fibre Toxicol..

[5] Zhao L., Zhu Y., Chen Z., Xu H., Zhou J., Tang S., Xu Z., Kong F., Li X., Zhang Y. (2018). Cardiopulmonary effects induced by occupational exposure to titanium dioxide nanoparticles. Nanotoxicology.

[6] Song B., Zhou T., Yang W., Liu J., Shao L. (2016). Contribution of oxidative stress to TiO_2_ nanoparticle-induced toxicity. Environ. Toxicol. Pharmacol..

[7] Chen T., Yan J., Li Y. (2014). Genotoxicity of titanium dioxide nanoparticles. J. Food Drug Anal..

[8] (2010). Carbon black, titanium dioxide, and talc. IARC Monogr Eval Carcinog Risks Hum..

[9] Ziental D., Czarczynska-Goslinska B., Mlynarczyk D.T., Glowacka-Sobotta A., Stanisz B., Goslinski T., Sobotta L. (2020). Titanium Dioxide Nanoparticles: Prospects and Applications in Medicine. Nanomaterials.

[10] Ursini C.L., Cavallo D., Fresegna A.M., Ciervo A., Maiello R., Tassone P., Buresti G., Casciardi S., Iavicoli S. (2014). Evaluation of cytotoxic, genotoxic and inflammatory response in human alveolar and bronchial epithelial cells exposed to titanium dioxide nanoparticles. J. Appl. Toxicol..

[11] Brandao F., Fernandez-Bertolez N., Rosario F., Bessa M.J., Fraga S., Pasaro E., Teixeira J.P., Laffon B., Valdiglesias V., Costa C. (2020). Genotoxicity of TiO_2_ Nanoparticles in Four Different Human Cell Lines (A549, HEPG2, A172 and SH-SY5Y). Nanomaterials.

[12] Tolliver L.M., Holl N.J., Hou F.Y.S., Lee H.J., Cambre M.H., Huang Y.W. (2020). Differential Cytotoxicity Induced by Transition Metal Oxide Nanoparticles is a Function of Cell Killing and Suppression of Cell Proliferation. Int. J. Mol. Sci..

[13] Baranowska-Wojcik E., Szwajgier D., Oleszczuk P., Winiarska-Mieczan A. (2020). Effects of Titanium Dioxide Nanoparticles Exposure on Human Health-a Review. Biol. Trace Elem. Res..

[14] Salou S., Cirtiu C.M., Lariviere D., Fleury N. (2020). Assessment of strategies for the formation of stable suspensions of titanium dioxide nanoparticles in aqueous media suitable for the analysis of biological fluids. Anal. Bioanal Chem..

[15] Rosario F., Bessa M.J., Brandao F., Costa C., Lopes C.B., Estrada A.C., Tavares D.S., Teixeira J.P., Reis A.T. (2020). Unravelling the Potential Cytotoxic Effects of Metal Oxide Nanoparticles and Metal(Loid) Mixtures on A549 Human Cell Line. Nanomaterials.

[16] Suzuki T., Miura N., Hojo R., Yanagiba Y., Suda M., Hasegawa T., Miyagawa M., Wang R.S. (2020). Genotoxicity assessment of titanium dioxide nanoparticle accumulation of 90 days in the liver of gpt delta transgenic mice. Genes Environ..

[17] Kazimirova A., El Yamani N., Rubio L., Garcia-Rodriguez A., Barancokova M., Marcos R., Dusinska M. (2020). Effects of Titanium Dioxide Nanoparticles on the Hprt Gene Mutations in V79 Hamster Cells. Nanomaterials.

[18] Oh N., Park J.H. (2014). Endocytosis and exocytosis of nanoparticles in mammalian cells. Int. J. Nanomed..

[19] Chen Z., Zhou D., Han S., Zhou S., Jia G. (2019). Hepatotoxicity and the role of the gut-liver axis in rats after oral administration of titanium dioxide nanoparticles. Part. Fibre Toxicol..

[20] Biola-Clier M., Gaillard J.C., Rabilloud T., Armengaud J., Carriere M. (2020). Titanium Dioxide Nanoparticles Alter the Cellular Phosphoproteome in A549 Cells. Nanomaterials.

[21] Liou S.H., Wu W.T., Liao H.Y., Chen C.Y., Tsai C.Y., Jung W.T., Lee H.L. (2017). Global DNA methylation and oxidative stress biomarkers in workers exposed to metal oxide nanoparticles. J. Hazard. Mater..

[22] Danielsen P.H., Knudsen K.B., Strancar J., Umek P., Koklic T., Garvas M., Vanhala E., Savukoski S., Ding Y., Madsen A.M. (2020). Effects of physicochemical properties of TiO_2_ nanomaterials for pulmonary inflammation, acute phase response and alveolar proteinosis in intratracheally exposed mice. Toxicol. Appl. Pharmacol..

[23] Pelclova D., Navratil T., Kacerova T., Zamostna B., Fenclova Z., Vlckova S., Kacer P. (2019). NanoTiO_2_ Sunscreen Does Not Prevent Systemic Oxidative Stress Caused by UV Radiation and a Minor Amount of NanoTiO_2_ is Absorbed in Humans. Nanomaterials.

[24] Uboldi C., Urban P., Gilliland D., Bajak E., Valsami-Jones E., Ponti J., Rossi F. (2016). Role of the crystalline form of titanium dioxide nanoparticles: Rutile, and not anatase, induces toxic effects in Balb/3T3 mouse fibroblasts. Toxicol. In Vitro.

[25] Vandebriel R.J., Vermeulen J.P., van Engelen L.B., de Jong B., Verhagen L.M., de la Fonteyne-Blankestijn L.J., Hoonakker M.E., de Jong W.H. (2018). The crystal structure of titanium dioxide nanoparticles influences immune activity in vitro and in vivo. Part. Fibre Toxicol..

[26] Yu Q., Wang H., Peng Q., Li Y., Liu Z., Li M. (2017). Different toxicity of anatase and rutile TiO_2_ nanoparticles on macrophages: Involvement of difference in affinity to proteins and phospholipids. J. Hazard. Mater..

[27] Ghosh M., Oner D., Duca R.C., Cokic S.M., Seys S., Kerkhofs S., Van Landuyt K., Hoet P., Godderis L. (2017). Cyto-genotoxic and DNA methylation changes induced by different crystal phases of TiO_2_-np in bronchial epithelial (16-HBE) cells. Mutat Res..

[28] Ursini C.L., Cavallo D., Fresegna A.M., Ciervo A., Maiello R., Buresti G., Casciardi S., Bellucci S., Iavicoli S. (2014). Differences in cytotoxic, genotoxic, and inflammatory response of bronchial and alveolar human lung epithelial cells to pristine and COOH– functionalized multiwalled carbon nanotubes. Biomed. Res. Int..

[29] Ursini C.L., Maiello R., Ciervo A., Fresegna A.M., Buresti G., Superti F., Marchetti M., Iavicoli S., Cavallo D. (2016). Evaluation of uptake, cytotoxicity and inflammatory effects in respiratory cells exposed to pristine and –OH and –COOH functionalized multi-wall carbon nanotubes. J. Appl. Toxicol..

[30] Cavallo D., Ciervo A., Fresegna A.M., Maiello R., Tassone P., Buresti G., Casciardi S., Iavicoli S., Ursini C.L. (2015). Investigation on cobalt-oxide nanoparticles cyto-genotoxicity and inflammatory response in two types of respiratory cells. J. Appl. Toxicol..

[31] Rietveld H.M. (1967). Line profiles of neutron powder-diffraction peaks for structure refinement. Acta Crystallogr..

[32] Rietveld H.M. (1969). A profile refinement method for nuclear and magnetic structures. J. Appl. Crystallogr..

[33] Wilhelmi V., Fischer U., van Berlo D., Schulze-Osthoff K., Schins R.P., Albrecht C. (2012). Evaluation of apoptosis induced by nanoparticles and fine particles in RAW 264.7 macrophages: Facts and artefacts. Toxicol. In Vitro.

[34] Guadagnini R., Halamoda Kenzaoui B., Walker L., Pojana G., Magdolenova Z., Bilanicova D., Saunders M., Juillerat-Jeanneret L., Marcomini A., Huk A. (2015). Toxicity screenings of nanomaterials: Challenges due to interference with assay processes and components of classic in vitro tests. Nanotoxicology.

[35] Collins A., El Yamani N., Dusinska M. (2017). Sensitive detection of DNA oxidation damage induced by nanomaterials. Free Radic Biol. Med..

[36] Ursini C.L., Cavallo D., Fresegna A.M., Ciervo A., Maiello R., Buresti G., Casciardi S., Tombolini F., Bellucci S., Iavicoli S. (2012). Comparative cyto-genotoxicity assessment of functionalized and pristine multiwalled carbon nanotubes on human lung epithelial cells. Toxicol. In Vitro.

[37] Petersen E.J., Reipa V., Watson S.S., Stanley D.L., Rabb S.A., Nelson B.C. (2014). DNA damaging potential of photoactivated p25 titanium dioxide nanoparticles. Chem. Res. Toxicol..

[38] Veranth J.M., Cutler N.S., Kaser E.G., Reilly C.A., Yost G.S. (2008). Effects of cell type and culture media on Interleukin-6 secretion in response to environmental particles. Toxicol. In Vitro.

[39] Teeguarden J.G., Hinderliter P.M., Orr G., Thrall B.D., Pounds J.G. (2007). Particokinetics in vitro: Dosimetry considerations for in vitro nanoparticle toxicity assessments. Toxicol. Sci..

[40] Skuland T., Ovrevik J., Lag M., Refsnes M. (2014). Role of size and surface area for pro-inflammatory responses to silica nanoparticles in epithelial lung cells: Importance of exposure conditions. Toxicol. In Vitro.

[41] Fenoglio I., Greco G., Livraghi S., Fubini B. (2009). Non-UV-induced radical reactions at the surface of TiO_2_ nanoparticles that may trigger toxic responses. Chemistry.

[42] Magdolenova Z., Collins A., Kumar A., Dhawan A., Stone V., Dusinska M. (2014). Mechanisms of genotoxicity. A review of in vitro and in vivo studies with engineered nanoparticles. Nanotoxicology.

[43] Sayes C.M., Wahi R., Kurian P.A., Liu Y., West J.L., Ausman K.D., Warheit D.B., Colvin V.L. (2006). Correlating nanoscale titania structure with toxicity: A cytotoxicity and inflammatory response study with human dermal fibroblasts and human lung epithelial cells. Toxicol. Sci..

[44] De Matteis V., Cascione M., Brunetti V., Toma C.C., Rinaldi R. (2016). Toxicity assessment of anatase and rutile titanium dioxide nanoparticles: The role of degradation in different pH conditions and light exposure. Toxicol. In Vitro.

[45] Falck G.C., Lindberg H.K., Suhonen S., Vippola M., Vanhala E., Catalan J., Savolainen K., Norppa H. (2009). Genotoxic effects of nanosized and fine TiO_2_. Hum. Exp. Toxicol..

[46] Ursini C.L., Campopiano A., Fresegna A.M., Ciervo A., Cannizzaro A., Angelosanto F., Maiello R., Iavicoli S., Cavallo D. (2019). Alkaline earth silicate (AES) wools: Evaluation of potential cyto-genotoxic and inflammatory effects on human respiratory cells. Toxicol. In Vitro.

[47] Jugan M.L., Barillet S., Simon-Deckers A., Herlin-Boime N., Sauvaigo S., Douki T., Carriere M. (2012). Titanium dioxide nanoparticles exhibit genotoxicity and impair DNA repair activity in A549 cells. Nanotoxicology.

[48] Gualtieri M., Ovrevik J., Holme J.A., Perrone M.G., Bolzacchini E., Schwarze P.E., Camatini M. (2010). Differences in cytotoxicity versus pro-inflammatory potency of different PM fractions in human epithelial lung cells. Toxicol. In Vitro.

[49] Park E.J., Yi J., Chung K.H., Ryu D.Y., Choi J., Park K. (2008). Oxidative stress and apoptosis induced by titanium dioxide nanoparticles in cultured BEAS-2B cells. Toxicol. Lett..

[50] Shi Y., Wang F., He J., Yadav S., Wang H. (2010). Titanium dioxide nanoparticles cause apoptosis in BEAS-2B cells through the caspase 8/t-Bid-independent mitochondrial pathway. Toxicol. Lett..

